# Kinetic Studies on the Removal of Some Lanthanide Ions from Aqueous Solutions Using Amidoxime-Hydroxamic Acid Polymer

**DOI:** 10.1155/2018/4058503

**Published:** 2018-07-08

**Authors:** Fadi Alakhras

**Affiliations:** Department of Chemistry, College of Science, Imam Abdulrahman Bin Faisal University, P.O. Box 1982, Dammam 31441, Saudi Arabia

## Abstract

Lanthanide metal ions make distinctive and essential contributions to recent global proficiency. Extraction and reuse of these ions is of immense significance especially when the supply is restricted. In light of sorption technology, poly(amidoxime-hydroxamic) acid sorbents are synthesized and utilized for the removal of various lanthanide ions (La^3+^, Nd^3+^, Sm^3+^, Gd^3+^, and Tb^3+^) from aqueous solutions. The sorption speed of trivalent lanthanides (Ln^3+^) depending on the contact period is studied by a batch equilibrium method. The results reveal fast rates of metal ion uptake with highest percentage being achieved after 15–30 min. The interaction of poly(amidoxime-hydroxamic) acid sorbent with Ln^3+^ ions follows the pseudo-second-order kinetic model with a correlation coefficient *R*^2^ extremely high and close to unity. Intraparticle diffusion data provide three linear plots indicating that the sorption process is affected by two or more steps, and the intraparticle diffusion rate constants are raised among reduction of ionic radius of the studied lanthanides.

## 1. Introduction

Fast industrial progress throughout preceding years involved improvement and wide handling of special materials in diverse viable products. The handling of heavy metals in manufacturing introduces a huge amount of toxic metals into the atmosphere in addition to the aquatic and global surroundings [1]. Due to the certain physical and chemical features of rare earth elements (REEs), they have been used for particular applications in advanced technologies, such as special ceramics, organic synthesis, and their usage in equipment such as batteries, sensors, energy efficient lighting, nuclear technologies, and telecommunication [2–5]. REEs can be exploited in the production of alloys, magnets, catalysts, electric machines, security systems, medical applications, and in fertilizing perspectives [6–10].

In spite of the increasing concern raised by REEs, healthiness advantages versus poisonous impacts of these matters should be taken seriously. There are several environmental concerns allied with the production, processing, and utilization of REEs [11]. A few reports have accounted that the chemicals used in the refining process of REEs are involved in disease and occupational poisoning of local residents, water pollution, and farmland destruction. It was confirmed that REE's bioaccumulation through the food chain can cause diseases because of the contact with human beings even at trace concentration [12–14]. Therefore, rare earth metal ions including lanthanides in wastewaters are of main environmental interest and necessitate to be treated prior to their removal into environment.

Disposal of rare earth metal ions from large volumes of wastewaters needs a cost-effectual remediation expertise. Using novel extraction techniques having great effectiveness and biodegradability will certainly decrease expenses and environmental effects, permitting the rare earth elements to be handled very extensively as well as escalating the quantities which are regained via recovering [15]. Several techniques have been used for rare earth elements removal such as solvent extraction, organic and inorganic ion exchange, micellar ultrafiltration, chemical precipitation, solid-phase extraction, or chromatography. Chemical precipitation treatment methods need large storage facilities for the precipitated sludge and sometimes require additional treatment options. Ion exchange, which is expensive and sophisticated, would allow metallic ions recovery. Additionally, these methods are not amenable to large-scale treatment of contaminated ground water or drinking water. Purification of water contaminated with lanthanides requires the ability to selectively remove the lanthanides in the presence of other ions like Ca and Mg [16]. Therefore, it may be of value to design a chemically selective sorbent material (amidoxime-hydroxamic acid as in this study), capable of selectively sequestering the lanthanides from waste streams, ground water, and drinking water, without the need for any additional solvents or treatment processes. Adsorption process which is used in this study is highly economical and capable of removing contaminants even at trace level. The use of this technique in water and wastewater treatment due to its simplicity and low cost of operation, and its wide end-use is highly utilized [17–19].

Nevertheless, the performance of any adsorption process is exceedingly reliant on the selection of suitable medium. For the extraction of lanthanides from aqueous systems, different prospective sorbents are studied by several research groups [20–24]. It is confirmed that the sorption of lanthanides can be affected by the variation of medium's acidity, contact period, temperature, and sorbent amount. Additionally, polymers with certain chelating ligands have been used to remove valuable ionic species found in liquid systems. These chelating polymers have attractive properties including powerful capability, great selectivity, and quick removal rates of ionic species with high physical potency and stability [25–28].

In connection with our previous work [17], poly(amidoxime-hydroxamic) acid resins were used for the sorption of various lanthanide ions (La^3+^, Nd^3+^, Sm^3+^, Gd^3+^, and Tb^3+^) from liquid systems. The impacts of pH, exposure time, counter ion, and cross-linker were investigated. The results showed that 6 hours of agitation was sufficient to accomplish utmost metal-ion removal with maximum at pH 7. The data also specified that the percentage removal declined as the quantity of cross-linker enhanced.

To understand the influence of exposure time and the kinetics of adsorption of lanthanide ions by amidoxime-hydroxamic acid polymers, more investigation were done and presented in this article. Pseudo-first-order and pseudo-second-order kinetics were utilized for data analysis. Furthermore, the intraparticle diffusion model which is developed to inspect the mechanism of sorption for a solid-liquid removal process was applied as well.

## 2. Materials and Methods

### 2.1. Materials

Unless otherwise stated all chemical materials were attained from trade sources and were utilized as obtained. Acrylonitrile, 2,2,4-trimethyl pentane, and gelatine were acquired from BDH Chemicals, Ltd. (Poole, England). Benzoyl peroxide and ethylacrylate were bought from Fluka (Buchs, Switzerland). Hydroxylamine hydrochloride was purchased from Aldrich (Milwaukee, WI). The following lanthanide salts were also used as received with no more refinement: LaCl_3_·6H_2_O, NdCl_3_·6H_2_O, SmCl_3_·6H_2_O, and GdCl_3_·6H_2_O from Aldrich and TbCl_3_·6H_2_O from K&K Laboratories, Inc. (Jamaica, NY).

### 2.2. Preparation and Characterization of Adsorbent Material

**Scheme 1:**
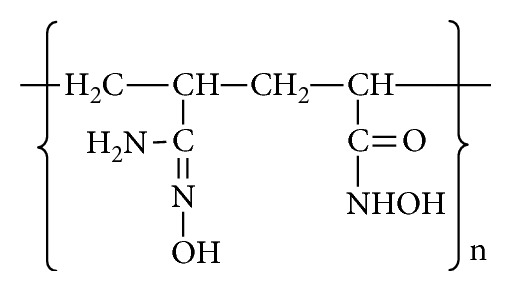
Structure of poly(amidoxime-hydroxamic) acid sorbent.

Amidoxime-hydroxamic acid polymer was synthesized and characterized depending on a process reported by Alakhras et al. [17]. The polymer (Scheme 1) was prepared through addition of acrylonitrile and ethylacrylate by suspension polymerization. Afterwards, hydroxylamine hydrochloride was added to convert the obtained copolymer into poly(amidoxime-hydroxamic) acid resin. A detailed analysis of IR spectra, decomposition point, and water regain parameters of the obtained chelating material was reported in our previous work [17].

### 2.3. Sorption Kinetics of Lanthanide Ions

A batch equilibrium process was employed to investigate the removal kinetics for each metal ion (La^3+^, Nd^3+^, Sm^3+^, Gd^3+^, and Tb^3+^). Perfectly weighted dosage of adsorbent (0.10 g) was constantly shaken at 400 rpm with 15 mL of acetic acid/acetate solution with pH 7.0 for two hours to reach equilibrium. After that, 10.0 mL of metal-ion solution with 15.0 mg·L^−1^ was added, and the mixture was shaken for a definite period of time. The agitation time was changed from 0.25 to 24 h. At the end, the contents of the flasks were determined for residual adsorbates by Complexometric titration with standard ethylene diamine tetra-acetic acid (EDTA) aqueous solution using xylenol orange as the indicator [29]. The flasks were shaken with a GFL-1083 thermostated shaker kept at 25°C.

The quantity of metal ions removed at equilibrium, *Q*_e_ (mg·g^−1^) was evaluated [30] according tothe following equation:

(1) $$\begin{array}{c} Q_{\text{e}}=\frac{\left(C_{0}-C_{\text{e}}\right)V}{W}, \end{array}$$

where *C*_0_ represents the initial metal amount (mg·L^−1^) and *C*_e_ is the final concentration after 24 h. The volume of solution (L) is represented by *V* and *W* symbolizes the amount of dry sorbent (g). The removal amount of metal ions chelated at several intervals [31] was computed by means of the following equation:

(2) $$\begin{array}{c} Q_{t}=\frac{\left(C_{0}-C_{t}\right)V}{W}, \end{array}$$

where *C*_*t*_ is the metal ion concentration (mg·L^−1^) in solution at different period *t*.

## 3. Results and Discussion

### 3.1. Sorption Kinetics

**Figure 1:**
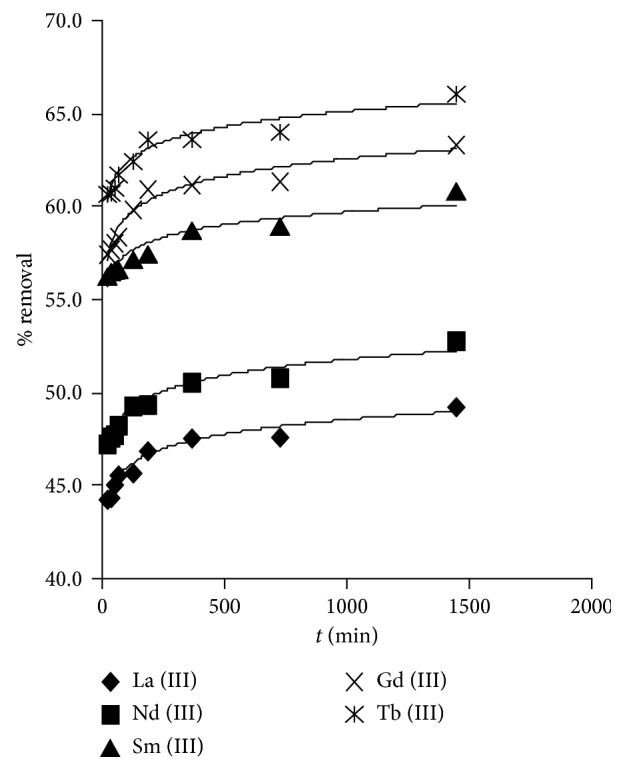
Effect of contact time on Ln^3+^ removal (*C*_0_ = 15 mg·L^−1^, *T* = 25°C, *W* = 0.10 g, and *V* = 0.025 L).

The removal rate of trivalent lanthanides (Ln^3+^) depending on the contact time was studied by a batch equilibrium method. In this procedure, 35–60 mesh size trials of the dry adsorbent was equilibrated for two hours with acetate solution (pH = 7.0).  Figure 1 demonstrates a representative reliance of lanthanides removal on exposure period. The percentage removal increased with time until it attained a steady state after 5-6 h. In addition, the outcome pointed out fast rates of ion sorption with 60% maximum percentage achieved after 15–30 min. This behaviour can be referred to the vacant active sites on the adsorbent surface and to the high electrostatic attraction between metal ions and chelating groups [32].

**Scheme 2:**
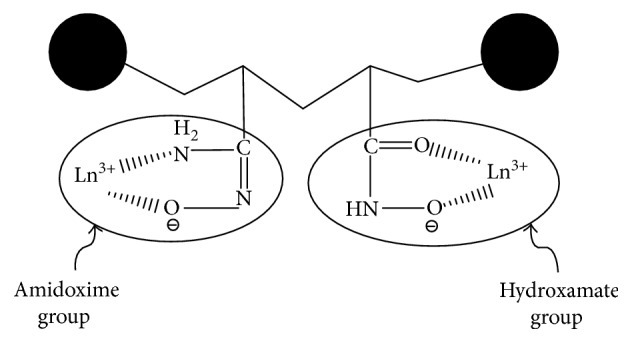
Lanthanide metal ions adsorption modes.

The above results can also be explained depending on metal ion adsorption modes (Scheme 2). Lanthanide metal ions can strongly attach via two sites (N and O) at the same time with different modes which certainly affects on the obtained complex stability.

Furthermore, results disclosed that the lanthanide ions removal follows the following arrangement: Tb^3+^ > Gd^3+^ > Sm^3+^ > Nd^3+^ > La^3+^; the polymer shows maximum removal ability toward Tb^3+^ and minimum for La^3+^. This divergence in capabilities depicted with the studied ions by the used polymer can be interpreted by the negative steric effect on coordination with active chelating groups [33]. The ionic radius for Tb^3+^ is 106.3 pm while 115 pm for La^3+^. The stability of the forming complex is anticipated to be less convenient for species of bigger extent; this is reliable with previous research [17, 34–36].

**Figure 2:**
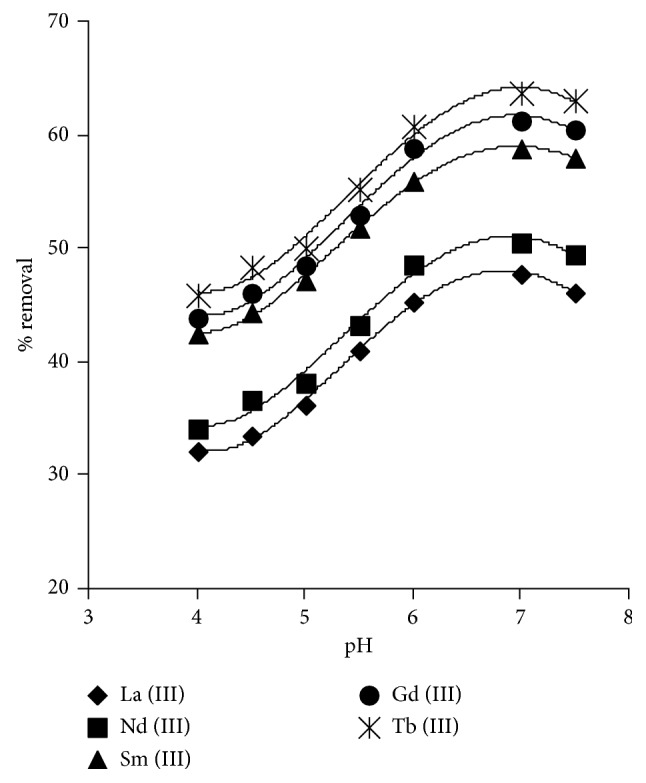
Effect of pH on Ln^3+^ removal (*C*_0_ = 15 mg·L^−1^, *T* = 25°C, *W* = 0.10 g, and *V* = 0.025 L).

The removal capacity of the investigated sorbent toward Ln^3+^ was investigated in the pH range 4.0–7.5 under continuous shaking for 24 h at 25°C. The effect of pH is depicted in Figure 2, and the results revealed that metal-ion uptake increased with increase of pH and a steady state was attained at about pH 7.0. This behaviour can be attributed to the acid dissociation nature of the hydroxamate and amidoxime groups of the sorbent material. At higher pH values, Ln^3+^ ions competed favourably toward donor sites compared with hydrogen ions. Consequently, more metal ions removal was achieved. However, at pH values higher than 7, the metal ion solution can be converted to hydroxide form that has no charge, and its interaction can be decreased clearly by the sorbent. The buffer capacity of acetic acid/acetate solution at pH 7.0 is still preserved and accepted under this condition in order to accomplish the highest percent removal of lanthanide metal ions using amidoxime-hydroxamic acid resin.

### 3.2. Kinetic Analysis of Lanthanide Ions Removal Method

To verify the kinetic phenomena of the removal process of lanthanide ions, three kinetic models were used to evaluate the experimental data at pH 7.0, involving pseudo-first-order, pseudo-second-order, and intraparticle diffusion models. The first two models described the whole sorption process without taking into account the diffusion steps, whereas the third model is utilized to investigate the mechanism of sorption for a solid-liquid removal process that can normally be depicted by three stages [15, 18, 32]. The representative equations for the kinetic models are offered in Table 1, where *Q*_*t*_ and *Q*_e_ are the quantity of ions removed at time *t* and at equilibrium in mg·g^−1^, correspondingly, *k* is the rate constant, and *C* is the boundary thickness layer in mg·g^−1^.

The (*Q*_e_)_cal_ and *k*_1_ data are presented in Table 2. Correlation coefficient *R*^2^ was varied between 0.7062 and 0.8964. The experimental *Q*_e_ values were considerably varied from the associated evaluated *Q*_e_ ones, demonstrating that the sorption process was not following the pseudo-first-order model.

**Figure 3:**
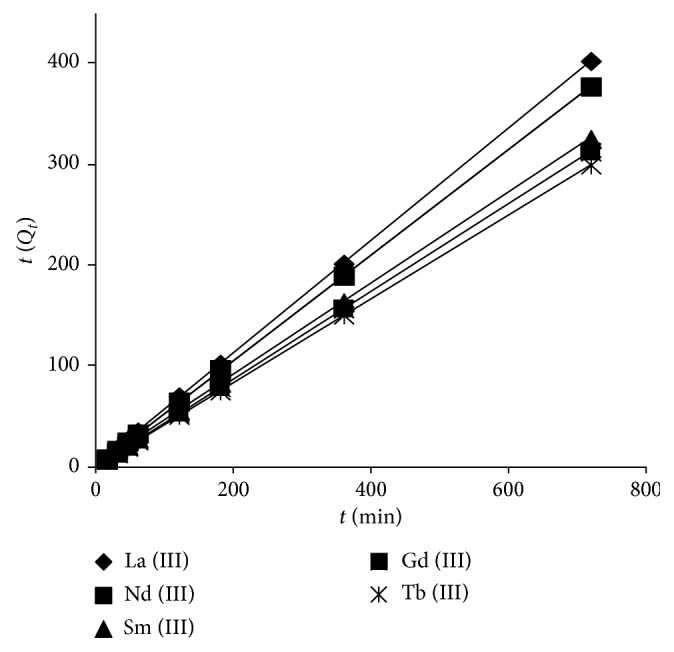
Pseudo-second-order kinetic plot for Ln^3+^ sorption.

Figure 3 displays plotting of *t*/*Q*_*t*_ against *t* for lanthanide metal ions under investigation with obtained straight lines having *R*^2^ = 0.9999. The (*Q*_e_)_cal_ data were in tremendous agreement with the investigational statistics (Table 2). Consequently, the Ln^3+^ removal process could be deduced more constructively by pseudo-second-order kinetic analysis. The outcome may propose that the removal of Ln^3+^ ions using amidoxime-hydroxamic acid polymer is based on a chemical adsorption process by means of participation or exchange of electrons between adsorbent and adsorbate [15, 37].

**Figure 4:**
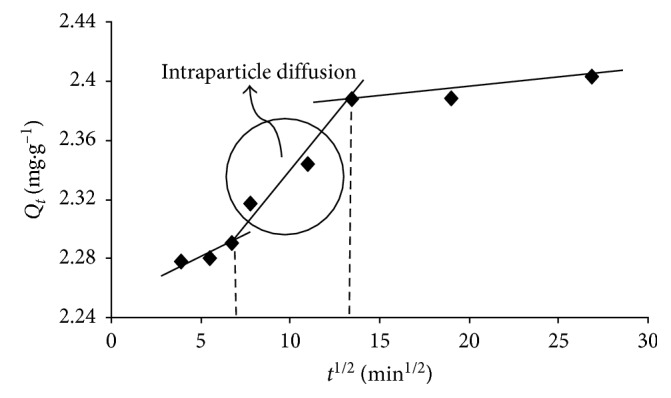
Intraparticle diffusion plot for the sorption of Tb(III) metal ion.

Intraparticle diffusion mechanism is described as the transfer of solute from liquid to solid phase during the removal process on a porous adsorbent. In this form, *k*_id_ (mg·g^−1^·min^−1/2^) is representing intraparticle diffusion rate constant while *C* (mg·g^−1^) is demonstrating the thickness of the boundary layer.  Figure 4 shows plotting of *Q*_*t*_ versus *t*^1/2^ for the sorption of Tb(III) metal ion. The data provided three linear plots indicating that the sorption process is affected by two or more steps. The first region characterized macropore diffusion, and the second one represented the slow removal phase, where intraparticle diffusion is the rate-limiting step [38, 39]. The third region could be considered as the diffusion via smaller pores, which is followed by the establishment of equilibrium. Additionally, the plot did not pass through the origin, demonstrating that pore diffusion could not be the only rate-determining step in verifying the kinetics of Ln^3+^ sorption process, and this process might be organized by a chemical reaction as well.

The values of the different parameters calculated from the plots of *Q*_*t*_ versus *t*^1/2^ for the intraparticle diffusion region are presented in Table 3.

**Figure 5:**
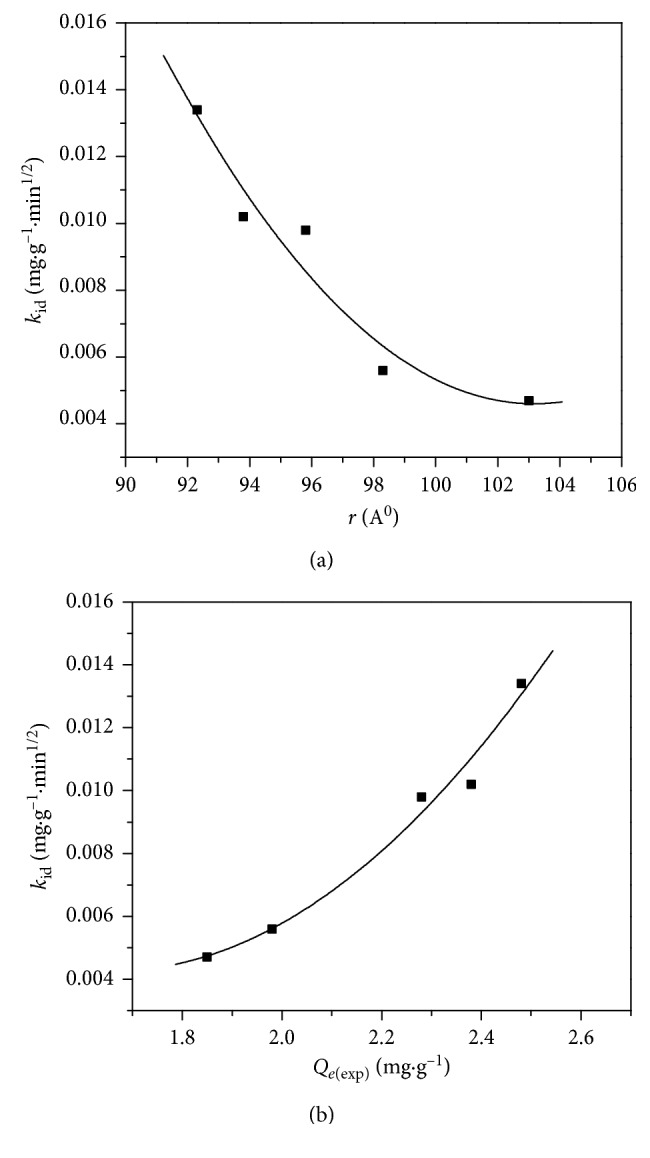
(a) Plot of ionic radius of Ln^3+^ versus *k*_id_ values; (b) plot of *Q*_e (exp)_ of Ln^3+^ versus *k*_id_ values.

It is clearly indicated that the intraparticle diffusion rate constants are increased with decrease of ionic radius of the studied lanthanides (Figure 5(a)). This behaviour suggested that ionic radius declined with the atomic number, and also smaller ions became more convenient to diffuse [15]. Furthermore, positive relationship was obtained by plotting *k*_id_ values versus experimental *Q*_e_ ones (Figure 5(b)). This performance designated that smaller metal ions can be diffused through the outer surface into the pores of the sorbent and removed more easily than bigger ones.

## 4. Conclusions

Amidoxime-hydroxamic acid polymer materials have been synthesized and exploited for the chelation of different lanthanides (La^3+^, Nd^3+^, Sm^3+^, Gd^3+^, and Tb^3+^) from aqueous solutions. The sorption rate of the investigated ions relying on agitation period was analyzed by the batch equilibrium method. The removal process was carried out with fast kinetic rates, and it achieved maximum percentage after 15–30 min. The sorption of ions followed the following order: Tb^3+^ > Gd^3+^ > Sm^3+^ > Nd^3+^ > La^3+^. Kinetic studies revealed that chemisorption was the rate-determining step with correlation coefficient remarkably high and close to unity. Intraparticle diffusion study afforded three regions demonstrating that the sorption course is affected by more than one step and intraparticle diffusion might be involving in controlling the diffusion of investigated lanthanides.

## Figures and Tables

**Table 1:** Linearized equations for Ln^3+^ sorption kinetics.

Kinetic model	Linear equation	Plot
Pseudo-first-order	ln (*Q*_e_ − *Q*_*t*_) = ln *Q*_e_ − *k*_1_*t*	ln (*Q*_e_ − *Q*_*t*_) versus *t*
Pseudo-second-order	(*t*/*Q*_*t*_)=(1/*k*_2_*Q*_e_^2^)+(*t*/*Q*_e_)	*t*/*Q*_e_ versus *t*
Intraparticle diffusion	*Q*_*t*_ = *k*_id_*t*^1/2^ + *C*	*Q*_*t*_ versus *t*^1/2^

**Table 2:** Kinetic constants for pseudo-first-order and pseudo-second-order models for Ln^3+^ sorption.

	(*Q*_e_)_exp_ (mg·g^−1^)	Pseudo-first-order			Pseudo-second-order		
		*K*_1_ (min^−1^)	(*Q*_e_)_cal_ (mg·g^−1^)	*R*_1_^2^	*K*_2_ (g·mg^−1^·min^−1^)	(*Q*_e_)_cal_ (mg·g^−1^)	*R*_2_^2^
La^3+^	1.85	1.61 × 10^−3^	0.1593	0.7754	0.1831	1.80	0.9999
Nd^3+^	1.98	1.38 × 10^−3^	0.1842	0.8457	0.1548	1.91	0.9998
Sm^3+^	2.28	1.38 × 10^−3^	0.1647	0.8964	0.1700	2.22	0.9999
Gd^3+^	2.38	1.61 × 10^−3^	0.1854	0.7062	0.1735	2.31	0.9997
Tb^3+^	2.48	1.38 × 10^−3^	0.1759	0.7304	0.1887	2.41	0.9999

**Table 3:** Intraparticle diffusion kinetic parameters for Ln^3+^ sorption.

Ln^3+^	*k*_id_ (mg·g^−1^·min^−1/2^)	*C* (mg·g^−1^)	*R* ^2^
La^3+^	0.0047	1.6228	0.9195
Nd^3+^	0.0056	1.7380	0.9536
Sm^3+^	0.0098	2.0938	0.9836
Gd^3+^	0.0102	2.1326	0.9603
Tb^3+^	0.0134	2.2314	0.9265

## Data Availability

The data used to support the findings of this study are available from the corresponding author upon request.
